# Inhalation of Nitrate of Silver, &c.

**Published:** 1849-08

**Authors:** Thomas K. Chambers

**Affiliations:** Fellow of the Royal College of Physicians


*Inhalation of Nitrate of Silver, &c. By* Thomas K. Chambers, m. d., Fellow of the Royal College of Physicians.—While treating diseases in those parts of the mucous membranes which are sufficiently exposed to sight and touch for the immediate application of remedial agents, there are few to whom the wish has not occurred, that equal facilities were afforded of directly influencing the deeper-seated continuations of the same fabric. The powerful remedies which restore so quickly to a healthy state the conjunctiva and the fauces, would probably act with equal rapidity and success on the stomach and bronchi, could we apply them rightly to the right spot, and attack the local disease without passing circuitously througn the whole system. A mode I have lately adopted of attaining this end with the most inaccessible mucous surface of all, the pulmonary, though it is clumsy and imperfect, may still be found useful in some obstinate cases, where the upper part of the air-tubes is the principal seat of disease.

The plan consists in the inhalation of a light innocuous powder, which may carry with it the required substance, either diffused in the air or absorbed in its pores. That which I have found well suited to the purpose is the pollen of the lycopodium or club-moss, which has been allowed to imbibe as much as it would take up of a saturated solution of nitrate of siiver, or of sulphate of copper, or the two combined, and then carefully dried, and reduced again to an impalpable powder. Mr. Squire has made me some which, in two grains and a half, contains one grain of nitrate of silver, and another which in five grains contains one of nitrate of silver and two of sulphate of copper. The patient should introduce into his mouth, as far as he can without choking, a well-dried glass funnel, and draw in his breath strongly, whilst he himself, or a second party, dusts the powder in a dense cloud into the large end with an ordinary nursery puff-ball. If the dust is raised by an attendant, the patient can indicate the moment he inspires by raising his hand.

To obviate the necessity for withdrawing the funnel after each inhalation, to prevent the dust being blown about the room, an apparatus with a double valve and a closed powder-box may be used, which allows the dust to pass inwards only; but the necessary employment of metal makes the machine less agreeable than the more awkward but cleaner-looking and less formidable glass.

There is usually a slight degree of coughing excited by the dusty vehicle, but not of such moment as to prevent an immediate repetition of the experiment. This is certainly an inconvenience, but it seems a much smaller one than that which attends the introduction of a sponge into the larynx, as has been recommended. The spasm excited by this is distressing to the operator and painful to the patient, and prevents its employment in slighter cases, where the remedy appears to both as bad as the disease. Moreover, the operation is a very dufficult one, requiring a rapid accuracy, a spirited tenderness of touch, as artists call it, which is the lot of few, and is seldom retained at that period of life when the intellect is most matured, but when the brush, the burin, and the scalpel, are handled with more judgment indeed, but with less elegance and delicacy.—*London Medical Gazette.*

